# The Solubility Parameters of Ionic Liquids

**DOI:** 10.3390/ijms11051973

**Published:** 2010-04-27

**Authors:** Andrzej Marciniak

**Affiliations:** Department of Physical Chemistry, Faculty of Chemistry, Warsaw University of Technology, Noakowskiego 3, 00-664 Warsaw, Poland; E-Mail: a.marciniak@ch.pw.edu.pl; Tel.: +48-222-345-816; Fax: +48-226-282-741

**Keywords:** ionic liquid, Hildebrand’s solubility parameter, standard enthalpy of vaporization

## Abstract

The Hildebrand’s solubility parameters have been calculated for 18 ionic liquids from the inverse gas chromatography measurements of the activity coefficients at infinite dilution. Retention data were used for the calculation. The solubility parameters are helpful for the prediction of the solubility in the binary solvent mixtures. From the solubility parameters, the standard enthalpies of vaporization of ionic liquids were estimated.

## 1. Introduction

Ionic liquids (ILs) have become the subject of an increasing number of investigations due to their unique properties such as wide liquid range, stability at high temperatures, no flammability and negligible vapor pressure. Ionic liquids as green solvents can be used in separation processes, synthesis, catalysis and electrochemistry, successfully replacing the conventional volatile, flammable and toxic organic solvents. Since the ILs have a negligible vapor pressure, the inverse gas chromatography (IGC) is a suitable method for measuring thermodynamic properties of pure substances and their mixtures [1]. From the retention data, the activity coefficients at infinite dilution, Flory-Huggins interaction parameters as well as the Hildebrand’s solubility parameters can be determined. Activity coefficients at infinite dilution are very important for calculations of selectivity and capacity of entrainers for the different separation problems, characterizing the behavior of liquid mixtures, estimation of mutual solubilities, fitting the excess molar energy (*G*^E^) model parameters (e.g., Wilson, NRTL, UNIQUAC), predicting the existence of an azeotrope, analytical chromatography, calculation of Henry constant and partition coefficients, development of thermodynamic models based on the group contribution methods such as mod. UNIFAC [2]. The values of the activity coefficients at infinite dilution for the investigated ionic liquids were published earlier [3–18].

The Hildebrand’s solubility parameters have numerous applications including gas-liquid solubility, solvent extraction and many others as described in detail in the literature [19,20]. Solubility parameters are available for only some of the ionic liquids determined by IGC [21–24], intrinsic viscosity method [25] or estimated from Kamlet-Taft equation [26]. This paper provides information on the Hildebrand’s solubility parameters determined for 18 ionic liquids as a function of temperature and the standard enthalpies of vaporization calculated from the values of the solubility parameters.

## 2. Results and Discussion

The Hildebrand’s solubility parameters (*δ*_2_) were calculated for the ionic liquids presented (with abbreviations and structures) in Table 1. The solubility parameters show a slight dependence on the temperature, which was also observed by Mutelet *et al.* [21–23]. The results are presented in Table 2 and are compared to results taken from the literature [21–26].

**Figure 1.**
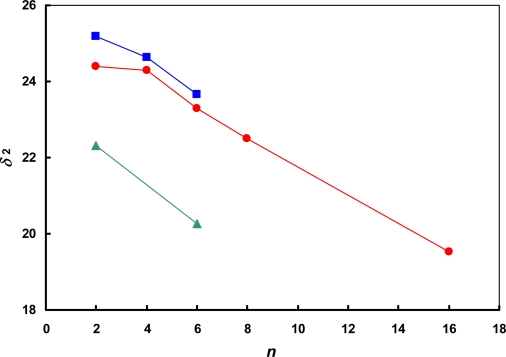
The solubility parameter *versus* the number of carbon atoms *n* in the alkyl chain R for the ionic liquids based on 1-alkyl-3-methyl-imidazolium cation [Rmim]^+^ obtained by IGC method. (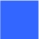) [SCN]^−^; (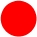) [BF_4_]^−^; (
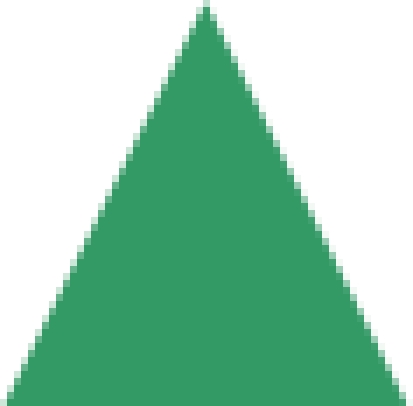
) [NTf_2_]^−^. The lines are drawn to guide the eye.

The values of *δ*_2_ calculated using the IGC method are not consistent with those obtained by the intrinsic viscosity method or estimated from the Kamlet-Taft equation. For ionic liquid [bmim][CF_3_SO_3_] the values of *δ*_2_ are 22.67, 24.9 [25] and 25.4 [26] obtained by IGC, intrinsic viscosity method or estimated from Kamlet-Taft equation, respectively. For ionic liquid [hmim][NTf_2_] the difference is much greater, values of *δ*_2_ are 20.25 and 25.6 [25] for the IGC and intrinsic viscosity methods, respectively. It was found that values of *δ*_2_ determined using the IGC method by Mutelet *et al.* [21–23] and Foco *et al.* [24] are also not consistent with those determined by the two methods mentioned above (Table 2). On the other hand, values obtained by different research groups by IGC are coherent as is shown in Figure 1. From Figure 1, it is obvious that for an ionic liquid of general cation formula [Rmim]^+^, the solubility parameter decreases with an increasing of the alkyl chain R. In the other words, the more aliphatic the cation character, the lower the solubility parameter. The slope of all three lines is similar – it confirms that the data are consistent (except for [emim][BF_4_] ionic liquid).

**Figure 2.**
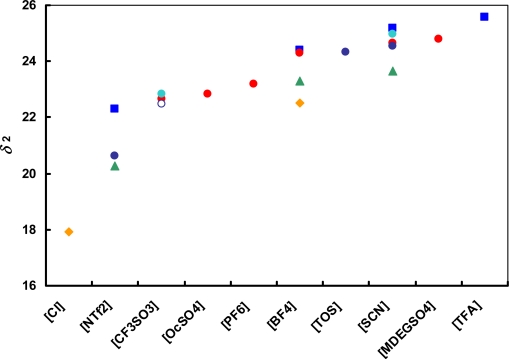
Anion influence on the solubility parameter for ionic liquids based on 1-alkyl-3-methyl imidazolium cations [Rmim]^+^, [bmPY]^+^ and [bmPYR]^+^ cations. (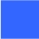) [emim]^+^; (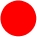) [bmim]^+^; (
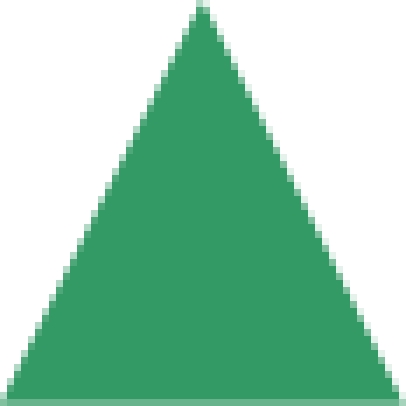
) [hmim]^+^; (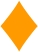) [omim]^+^; (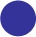) [1,4bmPY]^+^; (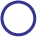) [1,3bmPY]^+^; (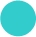) [bmPYR]^+^.

Figure 2 shows the anion influence on the solubility parameter for ionic liquids based on 1-alkyl-3-methyl-imidazolium cations [Rmim]^+^, 1-butyl-(3 or 4)-methyl-pyridinium [bmPY]^+^ and 1-butyl-1-methyl-pyrrolidinium [bmPYR]^+^ cations. The solubility parameter increases in the following order: [Cl]^−^ < [NTf_2_]^−^ < [CF_3_SO_3_]^−^ < [OcSO_4_]^−^ < [PF_6_]^−^ < [BF_4_]^−^ < [TOS]^−^ < [SCN]^−^ < [MDEGSO_4_]^−^ < [TFA]^−^. The highest values of *δ*_2_ are for [BF_4_]^−^, [TOS]^−^, [SCN]^−^, [MDEGSO_4_]^−^ and [TFA]^−^ anions, whilst the lowest value is for the [Cl]^−^ anion.

**Figure 3.**
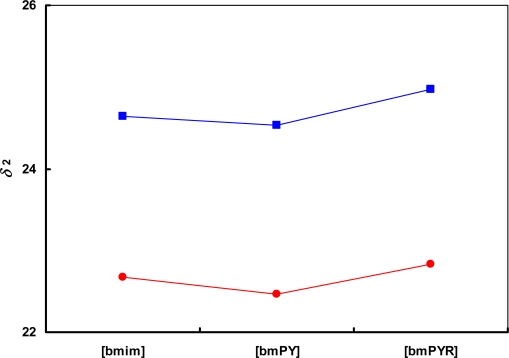
Influence of cation structure on the solubility parameter for ionic liquids based on (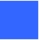) [SCN]^−^ and (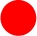) [CF_3_SO_3_]^−^ anions. The lines are drawn to guide the eye.

Figure 3 shows influence of the cation structure on the solubility parameter for ionic liquids based on [SCN]^−^ and [CF_3_SO_3_]^−^ anions. The lowest values of *δ*_2_ are for butyl-methyl-pyridinium [bmPY]^+^ cations ([1,3bmPY][CF_3_SO_3_] and [1,4bmPY][SCN]).

**Figure 4.**
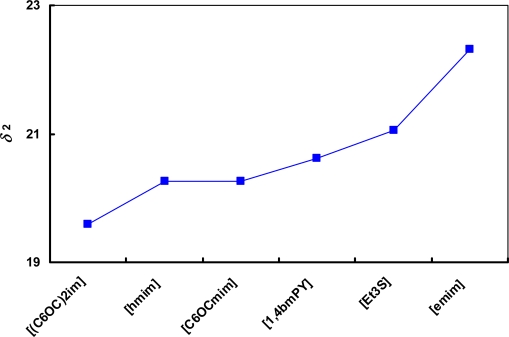
Cation influence on the solubility parameter for ionic liquids based on [NTf_2_]^−^ anion. The line is drawn to guide the eye.

The influence of the cation on the solubility parameter for the bis(trifluoromethylsulfonyl)-amide based ionic liquids ([NTf_2_]^−^) is shown in Figure 4. The solubility parameter increases in the following order: [(C_6_OC)_2_im]^+^ < [hmim]^+^ < [C_6_OCmim]^+^ < [1,4bmPY]^+^ < [Et_3_S]^+^ < [emim]^+^. The difference in solubility parameters between [hmim]^+^ and [C_6_OCmim]^+^ cations are very small. It is caused by the similar structure of these two cations. The [C_6_OCmim]^+^ cation has an additional methoxy group (–O–CH_2_–) in the structure, which causes a little augmentation of *δ*_2_ value. From this figure, it can be concluded again that the solubility parameter is higher for the ionic liquids with less aliphatic character. It is also presented in Figure 1 and was mentioned previously.

Standard enthalpies of vaporization Δ_vap_*H*_298.15_ calculated according to equation 8 and molar volumes of ionic liquids necessary in enthalpy calculations are presented in Table 3, and are contrasted the results taken from the literature [25–29]. The larger differences in values of enthalpies of vaporization are for ionic liquids based on the [SCN]^−^ anion. For ionic [bmim][CF_3_SO_3_] the difference is not so high: 22 and 13 kJ·mol^−1^ according to references [27] and [28], respectively. Due to the difference in solubility parameters, values of the enthalpies of vaporization calculated from data from references [25,26] are of course different and larger. For ionic liquid [1,4bmPY][NTf_2_] value of the enthalpy of vaporization is lower by 20 kJ·mol^−1^ than for that obtained by Deyko *et al.* [27]. A very good consistency in results of enthalpies of vaporization is found for [hmim][NTf_2_] ionic liquid. Result obtained from IGC measurements is only of about 2 and 4 kJ·mol^−1^ lower than for that obtained by Deyko *et al.* [27] and Zaitsau *et al.* [29], whilst the enthalpy of vaporization obtained from the solubility parameter determined by intrinsic viscosity method is much higher at of 216.4 kJ·mol^−1^ [25].

## 3. Calculation of Solubility Parameters

### 3.1. Experimental Procedure

The activity coefficients at infinite dilution for all investigated ionic liquids were measured using inverse gas chromatography. Detailed descriptions of materials, apparatus and methods used in each experiment are presented in the certain papers [3–18]. On the basis of the experimental data from the activity coefficients at infinite dilution measurements, the Hildebrand’s solubility parameters have been calculated using equations presented below.

### 3.2. Theoretical Basis

Retention data were used for the calculation of Hildebrand’s solubility parameters, *δ*_2_. According to the Flory-Huggins theory the interaction parameter at infinite dilution can be determined using the following expression:

(1) $$\chi_{12}^{\infty }=\text{ln}\left(\frac{273.15R}{P_{1}^{*}V_{g}M_{1}}\right)-\frac{P_{1}^{*}\left(B_{11}-V_{1}^{*}\right)}{RT}+\text{ln}\left(\frac{\rho_{1}}{\rho_{2}}\right)-\left(1-\frac{V_{1}^{*}}{V_{2}^{*}}\right)$$

where *R* denotes the gas constant, *T* the temperature, *P*_1_^*^ the saturated vapor pressure of the solute at temperature *T*, *B*_11_ the second virial coefficient of pure solute, *V*_1_^*^ and *V*_2_^*^ the molar volume of the solute and solvent respectively, *M*_1_ the molar mass of solute, *ρ*_1_ and *ρ*_2_ density of solute and solvent respectively, *V_g_* specific retention volume which is given by:

(2) $$V_{g}=\frac{273.15V_{\text{N}}}{Tm_{2}}$$

where *m*_2_ denotes the mass of the solvent on the column packing and *V*_N_ the net retention volume of the solute given by:

(3) $$V_{\text{N}}=J_{2}^{3}U_{\text{o}}(t_{\text{R}}-t_{\text{G}})$$

where *t*_R_ and *t*_G_ are the retention times for the solute and an unretained gas, respectively, *U*_o_ is the column outlet flow rate, $J_{2}^{3}$ the pressure correction term given by:

(4) $$J_{2}^{3}=\frac{2}{3}\frac{{\left(P_{\text{i}}/P_{\text{o}}\right)}^{3}-1}{{\left(P_{\text{i}}/P_{\text{o}}\right)}^{2}-1}$$

where *P*_i_ and *P*_o_ denote the inlet and the outlet pressure, respectively.

The column outlet flow rate corrected for the vapor pressure of water *U*_o_ is given by:

(5) $$U_{\text{o}}=U\left(1-\frac{P_{w}}{P_{o}}\right)\frac{T}{T_{f}}$$

where *T_f_* is the temperature of the flow meter, *P_w_* is the vapor pressure of water at *T_f_* and *U* is the flow rate measured with the bubble flow meter.

The interaction parameter $\chi_{12}^{\infty }$ may be expressed as a function of *δ*_1_ and *δ*_2_ which denote the solubility parameters of the solute and of the solvent, respectively by:

(6) $$\chi_{12}^{\infty }=\frac{V_{1}^{*}{\left(\delta_{1}-\delta_{2}\right)}^{2}}{RT}$$

Equation 6 can be rewritten as:

(7) $$\left(\frac{\delta_{1}^{2}}{RT}-\frac{\chi_{12}^{\infty }}{V_{1}^{*}}\right)=\left(\frac{2\delta_{2}}{RT}\right)\delta_{1}-\frac{\delta_{2}^{2}}{RT}$$

The solubility parameters *δ*_1_ of the solutes were calculated using following equation:

(8) $$\delta^{2}=\frac{\Delta_{\text{vap}}H-RT}{\upsilon }$$

where Δ_vap_*H* denotes enthalpy of vaporization and *υ* the molar volume. Enthalpies of vaporization of solutes were taken from literature [35] and molar volumes were calculated from densities taken from literature [36]. The values of *B*_11_ were calculated using the McGlashan and Potter [37] equation for alkanes and Tsonopolous [38] equation for the rest of solvents. The vapor pressure values were calculated using equation and constants taken from the literature [36,39,40]. Critical data used to calculate *B*_11_ were obtained from literature [41,42].

**Figure 5.**
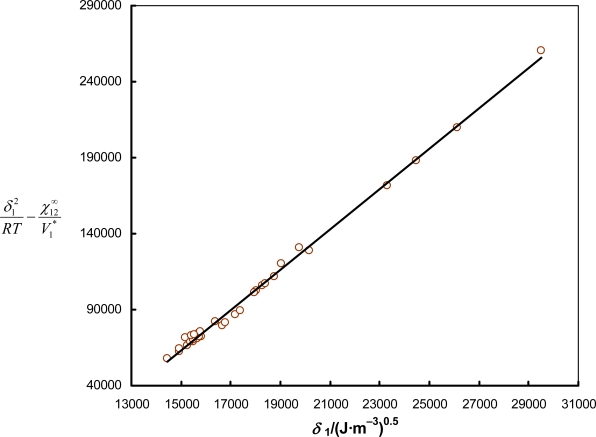
An example of the determination of solubility parameter *δ*_2_. Plot of $\frac{\delta_{1}^{2}}{RT}-\frac{\chi_{12}^{\infty }}{V_{1}^{*}}$ *versus* *δ*_1_ according to the equation 7 for ionic liquid [(C_6_OC)_2_im][NTf_2_] at *T* = 368.15 K.

Values of $\chi_{12}^{\infty }$ were determined from equation 1. If the left side of equation 7 is plotted against *δ*_1_, a straight line having a slope of 2*δ*_2_/*RT* and an intercept of $-\delta_{2}^{2}/RT$ is obtained. The solubility parameter of the solvent *δ*_2_ (ionic liquid) can be calculated from the slope and from the intercept of the straight line. The agreement of both *δ*_2_ values confirms the applicability of the method to the considered system. An example plot $\frac{\delta_{1}^{2}}{RT}-\frac{\chi_{12}^{\infty }}{V_{1}^{*}}$ *versus* *δ*_1_ is given in Figure 5 for ionic liquid [(C_6_OC)_2_im][NTf_2_] at *T* = 368.15 K. From the slope and interception of straight line the solubility parameter was determined, giving results of 20.30 and 20.40, respectively. Then the average of these values was taken as a final result. The correlation coefficient in this example is 0.996. Hildebrand’s solubility parameters of the investigated ionic liquids and the estimated standard enthalpy of vaporization calculated using equation 8 are listed in Tables 2 and 3, respectively.

## 4. Conclusions

Inverse gas chromatography is a reliable method to determine Hildebrand’s solubility parameters. Data obtained for 18 ionic liquids are coherent with those obtained by different research group by the same method. From the solubility parameters the standard enthalpies of vaporization can be calculated. Obtained values of enthalpies of vaporization are in acceptable consistency with the data available in literature except for ionic liquids based on thiocyanate anion.

## Figures and Tables

**Table 1.** Abbreviations, names and structures of investigated ionic liquids.

**Abbreviation**	**Name**	**Structure**	**Reference**
[emim][TFA]	1-Ethyl-3-methyl-imidazolium trifluoroacetate	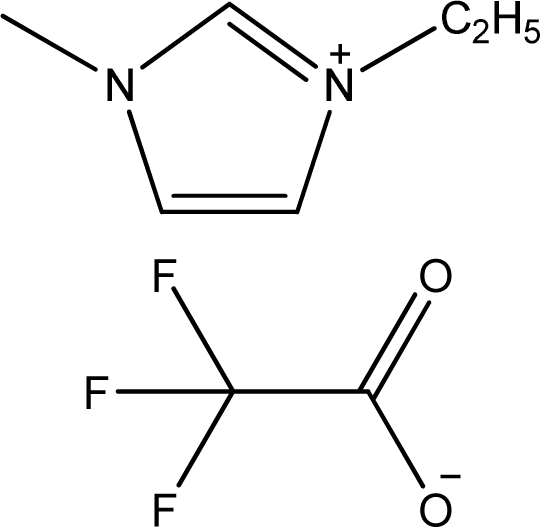	[3]
[emim][SCN]	1-Ethyl-3-methyl-imidazolium thiocyanate	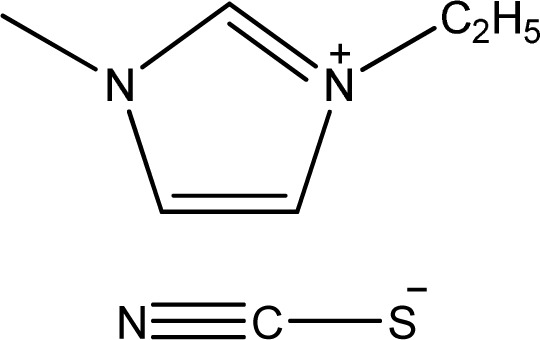	[4]
[bmim][SCN]	1-Butyl-3-methyl-imidazolium thiocyanate	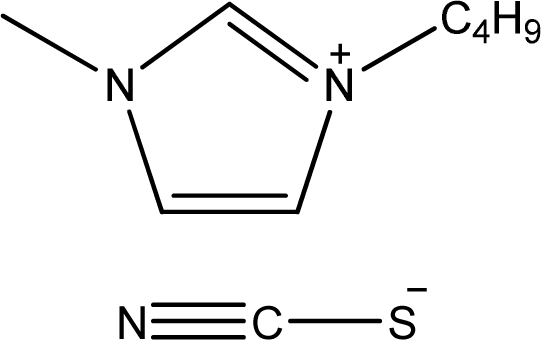	[5]
[hmim][SCN]	1-Hexyl-3-methyl-imidazolium thiocyanate	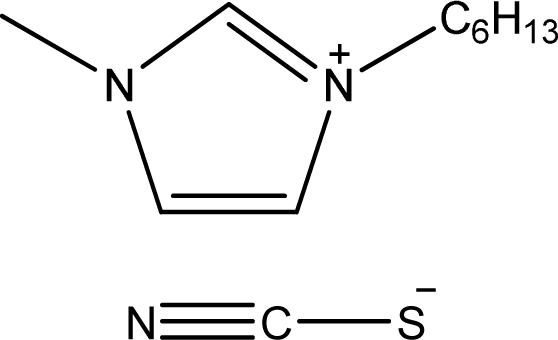	[6]
[1,4bmPY][SCN]	1-Butyl-4-methyl-pyridinium thiocyanate	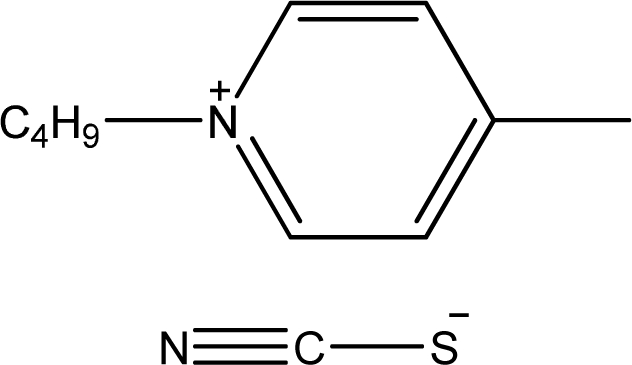	[7]
[bmPYR][SCN]	1-Butyl-1-methyl-pyrrolidinium thiocyanate	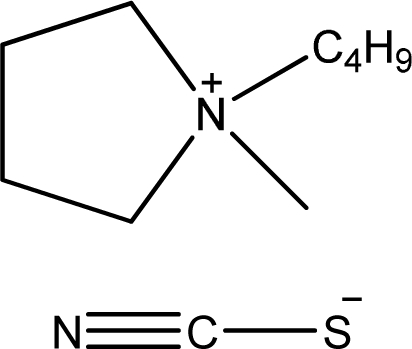	[7]
[bmim][CF_3_SO_3_]	1-Butyl-3-methyl-imidazolium trifluoromethanesulfonate	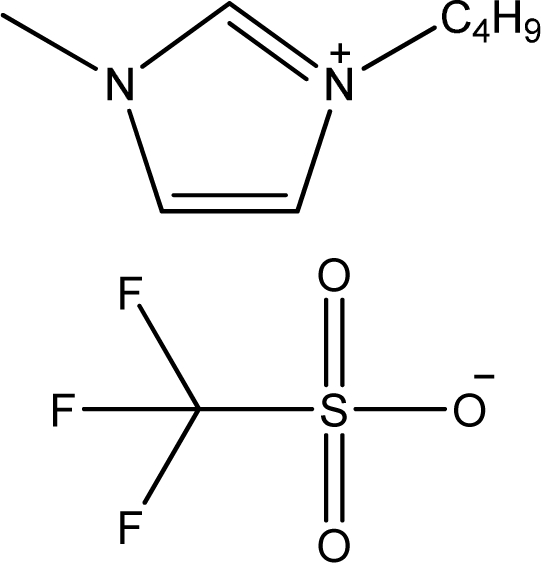	[8]
[1,3bmPY][CF_3_SO_3_]	1-Butyl-3-methyl-pyridinium trifluoromethanesulfonate	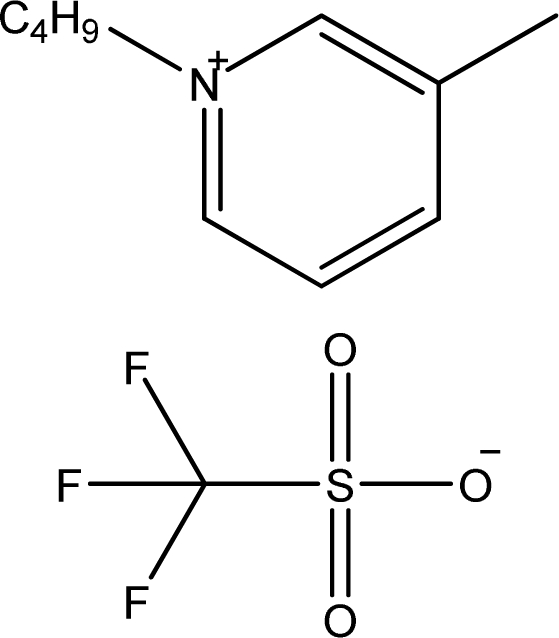	[9]
[bmPYR][CF_3_SO_3_]	1-Butyl-1-methyl-pyrrolidinium trifluoromethanesulfonate	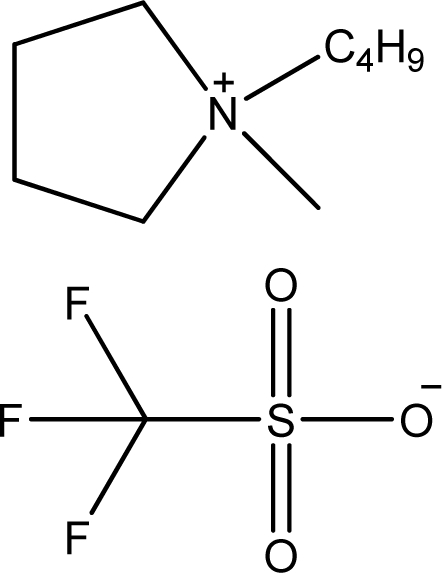	[10]
[bmim][MDEGSO_4_]	1-Butyl-3-methyl-imidazolium 2-(2-methoxyethoxy)ethyl sulfate	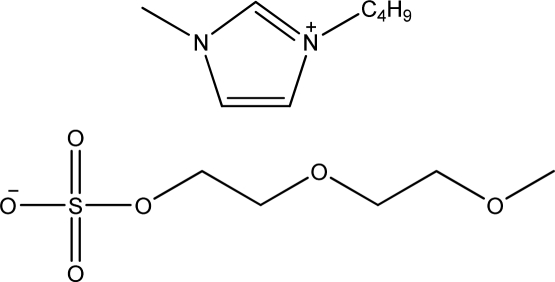	[11]
[bmim][OcSO_4_]	1-Butyl-3-methyl-imidazolium octyl sulfate	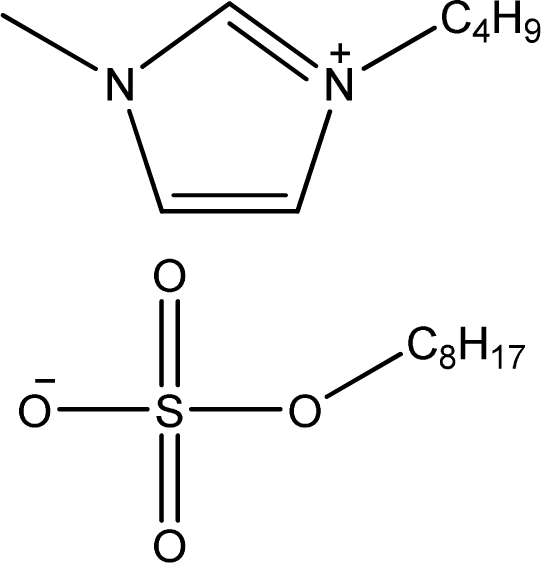	[12]
[P_1,i4,i4,i4_][TOS]	Triisobutyl-methyl-phosphonium tosylate	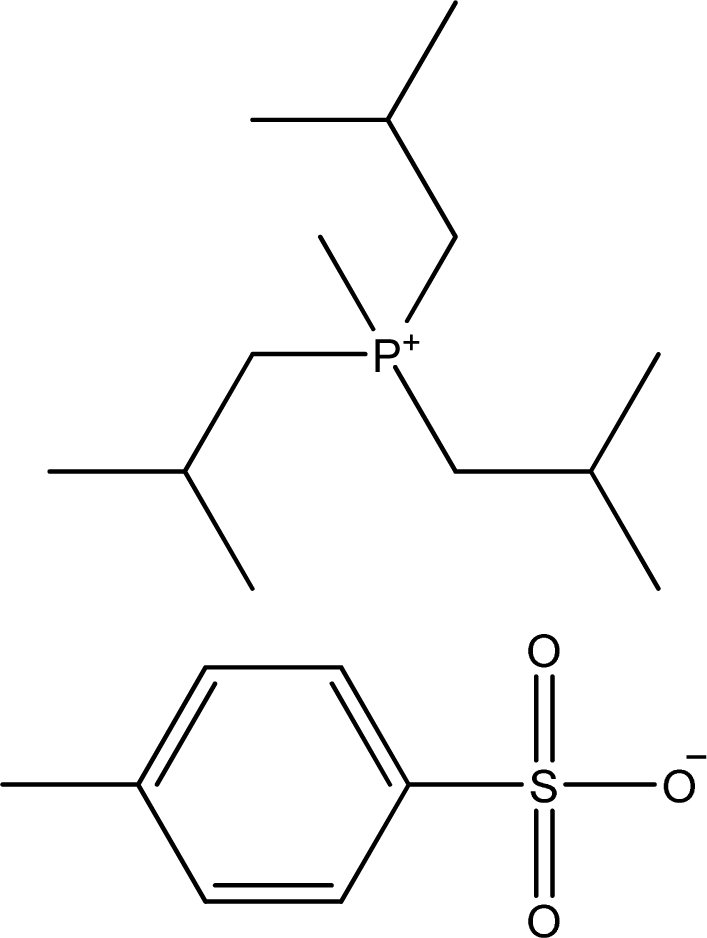	[13]
[1,4bmPY][TOS]	1-Butyl-4-methyl-pyridinium tosylate	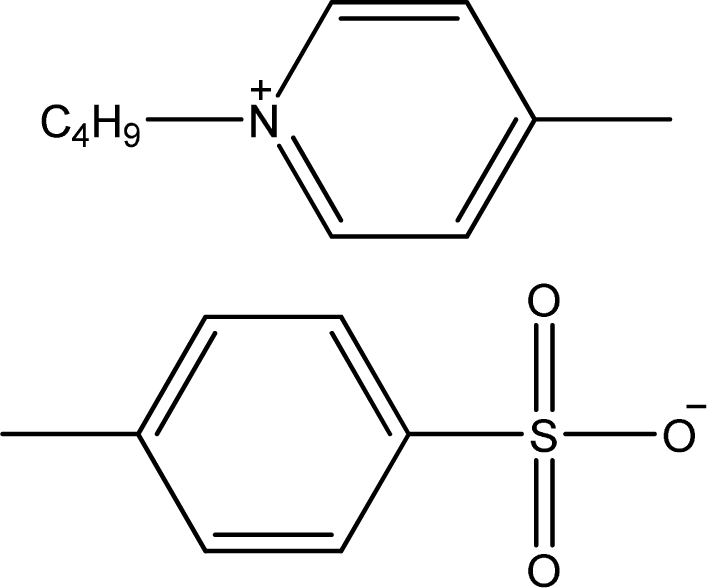	[14]
[1,4bmPY][NTf_2_]	1-Butyl-4-methyl-pyridinium bis(trifluoromethylsulfonyl)-amide	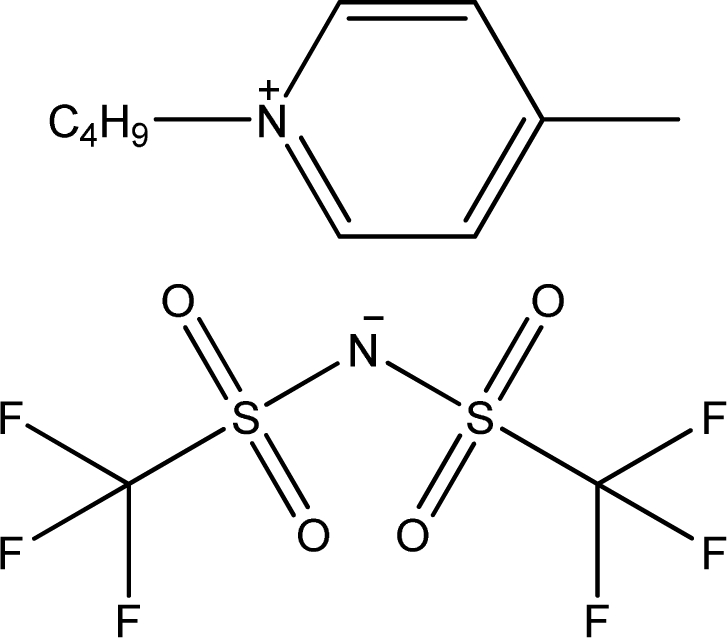	[15]
[C_6_OCmim][NTf_2_]	1-Hexyloxymethyl-3-methyl-imidazolium bis(trifluoromethylsulfonyl)-amide	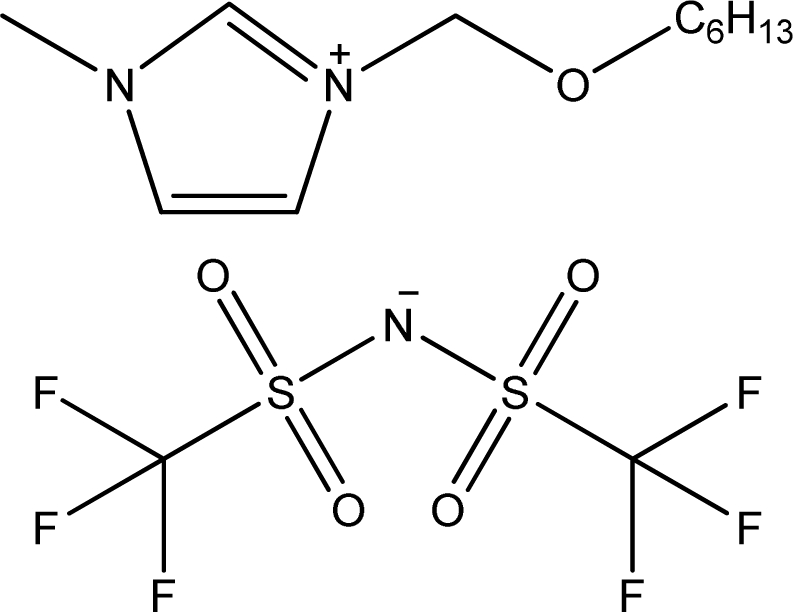	[16]
[(C_6_OC)_2_im][NTf_2_]	1,3-Dihexyloxymethyl-imidazolium bis(trifluoromethylsulfonyl)-amide	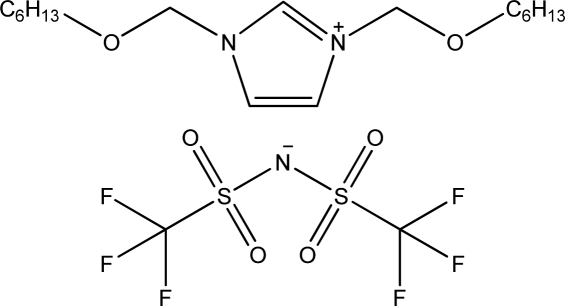	[16]
[Et_3_S][NTf_2_]	Triethyl-sulfonium bis(trifluoromethylsulfonyl)-amide	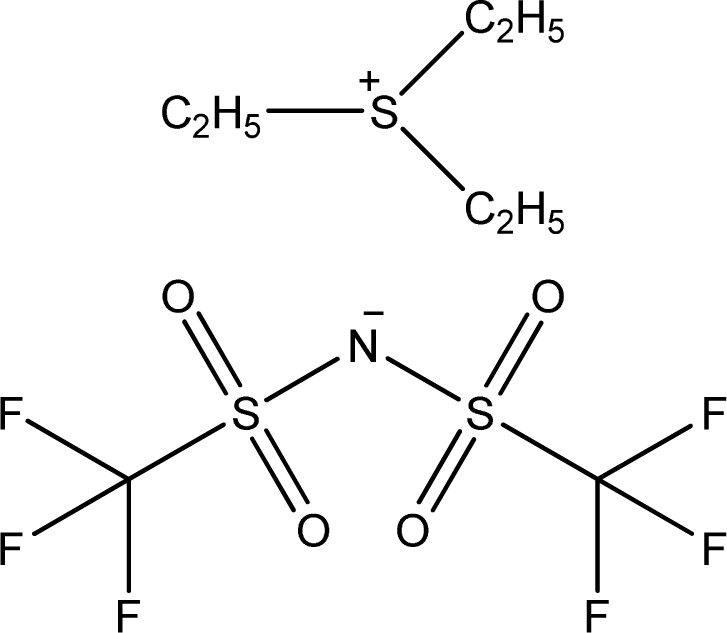	[17]
[hmim][NTf_2_]	1-Hexyl-3-methyl-imidazolium bis(trifluoromethylsulfonyl)-amide	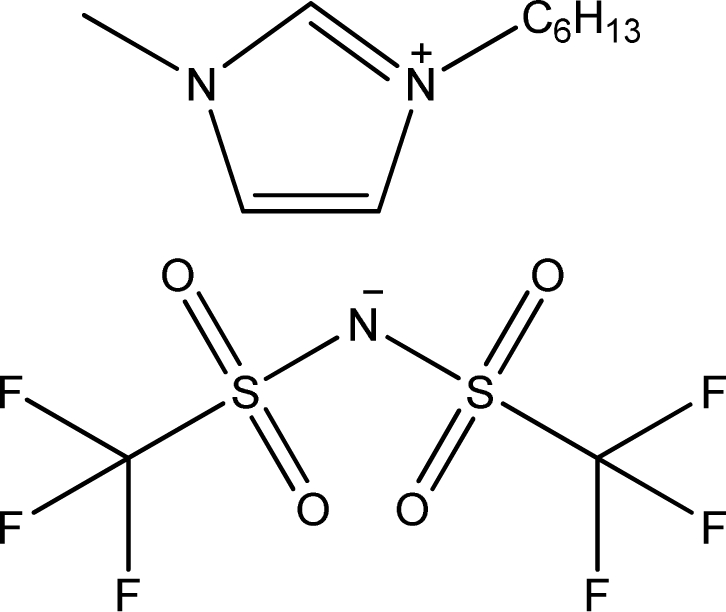	[18]

**Table 2.** Hildebrand’s solubility parameters *δ*_2_ for the different ionic liquids.

**Ionic liquid**	***T/K***	***δ*_2_/MPa^0.5^**
[emim][TFA]	298.15	25.56^1^
	328.15	25.58
	338.15	25.59
	348.15	25.60
	358.15	25.60

[emim][SCN]	298.15	25.19^1^
	308.15	25.24
	318.15	25.33
	328.15	25.41
	338.15	25.46
	348.15	25.55
	358.15	25.57

[bmim][SCN]	298.15	24.64^1^
	318.15	24.70
	328.15	24.72
	338.15	24.75
	348.15	24.77
	358.15	24.80

[hmim][SCN]	298.15	23.65^1^
	318.15	23.74
	328.15	23.79
	338.15	23.84
	348.15	23.90
	358.15	23.93
	368.15	23.98

[1,4bmPY][SCN]	298.15	24.53
	308.15	24.57
	318.15	24.62
	328.15	24.67
	338.15	24.71
	348.15	24.74
	358.15	24.77

[bmPYR][SCN]	298.15	24.96
	308.15	24.98
	318.15	25.00
	328.15	25.01
	338.15	25.02
	348.15	25.04
	358.15	25.05

[bmPYR][SCN]	298.15	24.96
	308.15	24.98
	318.15	25.00
	328.15	25.01
	338.15	25.02
	348.15	25.04
	358.15	25.05

[bmim][CF_3_SO_3_]	298.15	22.67^1^
	308.15	22.74
	318.15	22.81
	328.15	22.87
	338.15	22.97
	348.15	23.03
	358.15	23.09

[1,3bmPY][CF_3_SO_3_]	298.15	22.47^1^
	318.15	22.61
	328.15	22.68
	338.15	22.75
	348.15	22.84
	358.15	22.89

[bmPYR][CF_3_SO_3_]	298.15	22.83^1^
	318.15	22.94
	328.15	23.01
	338.15	23.06
	348.15	23.13
	358.15	23.17
	368.15	23.24

[bmim][MDEGSO_4_]	298.15	24.80
	303.15	24.80
	308.15	24.81

[bmim][OcSO_4_]	298.15	22.83
	313.15	23.00
	328.15	23.25

[P_1,i4,i4,i4_][TOS]	298.15	24.33^1^
	318.15	24.20
	328.15	24.13
	338.15	24.05
	348.15	23.99
	358.15	23.93

[1,4bmPY][TOS]	298.15	23.06^1^
	328.15	23.24
	333.15	23.27
	338.15	23.29
	343.15	23.33

[1,4bmPY][NTf_2_]	298.15	20.61^1^
	318.15	20.82
	328.15	20.92
	338.15	21.05
	348.15	21.15
	358.15	21.25
	368.15	21.35

[C_6_OCmim][NTf_2_]	298.15	20.26^1^
	318.15	20.48
	328.15	20.59
	338.15	20.71
	348.15	20.82
	358.15	20.93
	368.15	21.05

[(C_6_OC)_2_im][NTf_2_]	298.15	19.60^1^
	318.15	19.81
	328.15	19.92
	338.15	20.03
	348.15	20.14
	358.15	20.25
	368.15	20.35

[Et_3_S][NTf_2_]	298.15	21.05^1^
	308.15	21.13
	318.15	21.25
	328.15	21.35
	338.15	21.47
	348.15	21.55
	358.15	21.66
	368.15	21.72

[hmim][NTf_2_]	298.15	20.25
	308.15	20.36
	313.15	20.44
	328.15	20.58
	333.15	20.64
	338.15	20.70
	348.15	20.83

Solubility parameters taken from the literature		

[mmim][(CH_3_)_2_PO_4_] [21]	312.55	26.54
	332.65	25.96
	352.75	25.16

[emim][(C_2_H_5_)_2_PO_4_] [21]	312.65	25.81
	332.55	25.44
	352.65	25.32

[emim][NTf_2_] [23]	313.15	22.31

[emim][NTf_2_] [25]	298.15	27.6

[emim][BF_4_] [24]	298.15	24.4

[bmim][BF_4_] [24]	298.15	24.3

[bmim][BF_4_] [25]	298.15	31.6

[bmim][NTf_2_] [25]	298.15	26.7

[bmim][NTf_2_] [26]	298.15	25.5

[bmim][CF_3_SO_3_] [25]	298.15	24.9

[bmim][CF_3_SO_3_] [26]	298.15	25.4

[bmim][PF_6_] [23]	313.15	23.2
	323.15	22.62
	333.15	22.05

[bmim][PF_6_] [25]	298.15	29.8

[bmim][PF_6_] [26]	298.15	30.2

[bmim][SbF_6_] [26]	298.15	31.5

[bmmim][NTf_2_] [26]	298.15	24.2

[hmim][BF_4_] [24]	298.15	23.3

[hmim][NTf_2_] [25]	298.15	25.6

[hmim][PF_6_] [25]	298.15	28.6

[omim][BF_4_] [24]	298.15	22.5

[omim][NTf_2_] [25]	298.15	25.0

[omim][PF_6_] [25]	298.15	27.8

[omim][Cl] [23]	313.15	17.91

[C_16_mim][BF_4_] [22]	323.15	19.52
	333.15	19.61
	343.15	19.60

[OH-C_2_mim][BF_4_] [21]	302.55	22.77
	312.65	22.87
	332.65	22.88

[OH-C_2_mim][PF_6_] [21]	302.65	21.84
	312.55	21.93
	332.45	22.45

1 extrapolated values.

**Table 3.** Molar volumes *V_m_* at *T* = 298.15 K and standard enthalpies of vaporization Δ_vap_*H*_298.15_ for investigated ionic liquids.

**Ionic liquid**	***V*_*m*_/cm^3^·mol^−1^**	**Δ_vap_*H*_298.15_/kJ·mol^−1^**				
[emim][TFA]	173.7^1^	115.9^7^				
[emim][SCN]	151.6^2^	98.6^7^	151^8^			
[bmim][SCN]	184.4^3^	114.5^7^	148^8^			
[hmim][SCN]	200.0^4^	114.3^7^				
[1,4bmPY][SCN]	196.2^5^	120.5				
[bmPYR][SCN]	188.8^5^	120.1				
[bmim][CF_3_SO_3_]	222.0^5^	116.6^7^	139^8^	130.2^9^	140.1^10^	145.7^11^
[1,3bmPY][CF_3_SO_3_]	234.7^5^	121.0^7^				
[bmPYR][CF_3_SO_3_]	232.6^5^	123.7^7^				
[bmim][MDEGSO_4_]	284.2^5^	177.6				
[bmim][OcSO_4_]	327.7^5^	173.0				
[P_1,i4,i4,i4_][TOS]	363.4^6^	217.6^7^				
[1,4bmPY][NTf_2_]	304.8^5^	132.0^7^	152^8^			
[C_6_OCmim][NTf_2_]	349.9^5^	146.0^7^				
[(C_6_OC)_2_im][NTf_2_]	460.2^5^	179.2^7^				
[Et_3_S][NTf_2_]	273.7^5^	123.7^7^				
[hmim][NTf_2_]	326.4^5^	136.7	139^8^	141.6^12^	216.4^10^	

1 from reference [30];

2 from reference [31];

3 from reference [32];

4 from reference [33];

5 from density measurements performed on Anton Paar Density Meter DMA 4500;

6 from reference [34];

7 calculated from extrapolated values of *δ*_2_;

8 from reference [27];

9 from reference [28];

10 calculated from *δ*_2_ from reference [25];

11 calculated from *δ*_2_ from reference [26];

12 from reference [29]

## Electronic Supporting Information

Table 1S, interaction parameters, $\chi_{12}^{\infty }$.
